# Primary care chaplaincy: an intervention for complex presentation

**DOI:** 10.1017/S1463423618000737

**Published:** 2018-10-08

**Authors:** Gordon W. Macdonald

**Affiliations:** GP Principal, Regent Gardens Medical Practice, Glasgow, UK

**Keywords:** chaplaincy, isolation, presenting symptoms, undifferentiated, well-being

## Abstract

**Aim:**

To determine the responsiveness of primary care chaplaincy (PCC) to the current variety of presenting symptoms seen in primary care. This was done with a focus on complex and undifferentiated illness.

**Background:**

Current presentations to primary care are often complex, undifferentiated and display risk factors for social isolation and loneliness. These are frequently associated with loss of well-being and spiritual issues. PCC provides holistic care for such patients but its efficacy is unknown in presentations representative of such issues. There is therefore a need to assess the characteristics of those attending PCC. The effectiveness of PCC relative to the type and number of presenting symptoms should also be analysed whilst evaluating impact on GP workload.

**Methods:**

This was a retrospective observational study based on routinely collected data. In total, 164 patients attended PCC; 75 were co-prescribed antidepressants (AD) and 89 were not (No-AD). Pre- and post-PCC well-being was assessed by the Warwick–Edinburgh mental well-being score. Presenting issue(s) data were collected on a separate questionnaire. GP appointment utilisation was measured for three months pre- and post-PCC.

**Findings:**

Those displaying undifferentiated illness and risk factors for social isolation and loneliness accessed PCC. PCC (No-AD) was associated with a clinically meaningful and statistically significant improvement in well-being in all presenting issues. This effect was maintained in those with multiple presenting issues. PCC was associated with a reduction in GP appointment utilisation in those not co-prescribed AD.

## Introduction

In recent years we have witnessed the emergence of chaplaincy provision in primary care in the United Kingdom. This has arisen in response to several factors. It is acknowledged by GP’s, that spirituality occupies a central place within their sense of personhood (Appleby *et al*., 2018). Patients are often aware of their spiritual needs and the requirement for these to be addressed (Williams *et al*., 2011). However, clinicians can be reticent in raising such issues due to lack of experience or concern regarding non-engagement (Vermandere *et al*., 2011). In view of this it has been suggested that GP’s may act as spiritual generalists who refer to the specialist chaplain (Hamilton *et al*., 2017).

Defining spirituality in a healthcare setting can be challenging with each description presenting its own nuance, making a precise definition seem elusive. Such broad definitions are, however, useful in allowing provision of care to those of faith or no faith. Regardless of definitions the literature coalesces around certain key components of what spirituality entails: searching for meaning amidst suffering (Frankl, 1984); a search for the sacred (Pargament, 1999); and a sense of acceptance, being loved, retaining self-worth and a sense of integration and purpose (Hamilton *et al*., 2017). Such wholeness and peace may ultimately develop through ‘spiritual direction’ and being pointed to that which is transcendent and beyond oneself, which is one differentiating feature of spiritual care relative to other psychological therapies (Maslow, 1970; King and Koenig, 2009).

## Primary care chaplaincy (PCC)

Several approaches are taken in PCC to facilitate spiritual direction. Identifying signs of hope and moments of pleasure are used to promote positive direction (Bryson *et al*., 2012). Compassionate presence and listening with generosity allow time and space for questions regarding suffering and meaning (Mcsherry *et al*., 2016). Helman’s folk model of rhetorical ‘why, why me, what next’ type questions can be utilised by the chaplain to initiate steps towards meaning, acceptance and peace (Helman, 1981). In this study PCC is based on a ‘Human Givens’ approach (Griffen, 2004). The ‘Human Givens’ approach recognises our innate emotional needs as comprising of a need for security, significance and self-worth and that when these are not met there is a loss of well-being. PCC seeks to identify when these needs are unmet and does so by responding to symptoms of depression, anxiety and ‘modern maladies’. Such modern maladies arise in the prevailing philosophical milieu of reductionism, individualism and consumerism and are described as loss of well-being, obesity and addictions amongst others (Hanlon *et al*., 2011). These are seen as pointers to unmet human givens or spiritual needs and prompt referral into PCC.

PCC has taken two main forms: community chaplaincy listening (Mowat *et al*., 2012; Bunniss and Mowat, 2013) or this ‘Human Givens’ bio-psycho-social model utilised by several practices (Bryson *et al*., 2012; Macdonald, 2017b). Both iterations are gaining traction with service expansion and an increasing evidence base.

PCC has been shown to be associated with an improvement in well-being score (Kevern and Hill, 2015). This improvement remains present when it is used as the sole intervention [ie, excluding patients co-prescribed antidepressants (AD)] and is similar to the improvement in well-being seen with AD (Macdonald, 2017a). Perhaps more importantly the narrative feedback highlights the many valued aspects of PCC: empowerment, enhanced self-esteem and resilience (Mcsherry *et al*., 2016).

It may be that PCC has grown as a pragmatic response to the need for readily accessible ‘talking therapies’. As previously described, PCC may also provide a holistic intervention for those suffering from increasingly prevalent long-term conditions or ‘modern maladies’ such as loss of well-being, obesity or depression (Hanlon *et al*., 2011; Barnett *et al*., 2012).

## Undifferentiated illness

The role of the modern GP is described as that of the ‘expert medical generalist’ in the new Scottish GP contract (Robison and Mcdevitt, 2017). This is seen to include care of patients presenting with undifferentiated illness and the coordination of care for those with complex presentations/multiple needs. Undifferentiated illness is that which presents in a non-specific manner often at an early stage in the disease process making diagnosis more challenging (Shinkins and Perera, 2013). It is acknowledged that undifferentiated illness may be most prevalent in primary care (Alam *et al*., 2017). Spiritual needs frequently present in such a undifferentiated manner with patients finding it difficult to engage with or effectively articulate the deepest questions of their life (Puchalski, 2001). Patients attending PCC have a wide variety of symptoms triggered by a multifaceted interaction between the physical and the spiritual. Often these are expressed in an undifferentiated manner, that is in terms of loss of well-being as distinct but somewhat less tangible than depression or anxiety (Macdonald, 2017a). Such undifferentiated illnesses often present with more than one issue or symptom (Green and Holden, 2003), and this is the experience in PCC. This type of complexity or multimorbidity is well evidenced in primary care (Barnett *et al*., 2012). PCC will therefore need to be responsive to a wide variety of symptoms, multimorbidity and undifferentiated illness to remain relevant to the presentations seen in primary care.

## Current context

Social isolation (diminished social networks) and loneliness (a negative subjective experience of reduced social interaction) may contribute to the above drivers for PCC (Valtorta and Hanratty, 2012). Some research indicates an association with increased mortality rates and poorer health outcomes in the lonely (Bhatti and Haq, 2017). One review suggests loneliness has a similar impact to other well established risk factors such as obesity (Holt-Lunstad *et al*., 2010). An association between isolated or lonely patients and increased consultation rates has been seen in primary care (Ellaway *et al*., 1999). Furthermore a correlation between loneliness and reduced spiritual well-being has been suggested (Miller, 1985). This would seem to point to the need for the GP to be aware of the holistic care required for those who are lonely or isolated.

It was also well described by Pink *et al*., that due to the dominance of the reductionist/rationalistic western worldview over the spiritual, GPs perform a surrogate priestly role in presentations such as bereavement, loneliness and social isolation (Pink *et al*., 2007). The current chair of the RCGP used her inaugural conference speech to call for more time for lonely patients and additional members of the healthcare team to care for the lonely or isolated (Rimmer, 2017). It seems that PCC would fulfil this remit allowing the ‘priestly’ function of the GP to be somewhat divested.

## Rationale for study

It is likely that PCC is currently caring for those with undifferentiated and complex/multiple presenting symptoms. It is not known to what extent the presenting issue affects initial well-being. The impact of PCC in multiple issue presentations relative to single-issue presentations is also unknown.

It is possible that loneliness and social isolation are contributing to loss of well-being and those experiencing these are using primary care as a resource.

It is not known to what extent those who are socially isolated or lonely are attending PCC or if this can be interpreted from existing data. It is not the within the remit of this study to assess the efficacy of PCC in those with social isolation or loneliness as this was not directly tested for. However, it was felt useful to determine if those with risk factors for social isolation or loneliness were using PCC, as a starting point for further research.

The impact on GP workload in those patients attending PCC who are not on AD has not been evaluated. Workload has only been compared between PCC patients and those on AD.

There seems a need, therefore, to further study PCC in terms of initial presentations, complexity and workload. This will establish if this service is fit for the purpose of caring for the complex and undifferentiated presentations we see arising from our individualistic society.

## Objectives

- Assess the impact of presenting issues on the initial and change in well-being scores.
- Assess the impact of single relative to multiple presenting issues on the initial and change in well-being scores.
- Determine if PCC is being utilised by those with recognised risk factors for social isolation/loneliness.
- Determine effect on GP workload of patients attending PCC.

## Methods

### Study design

This was a retrospective service evaluation. The study was observational with no randomisation or allocation occurring. This study was a continuation of previously published data (Macdonald, 2017a), with data collection occurring between March 2015 and July 2017. This generated a further 11 months of data and allowed a more comprehensive analysis of above objectives.

### Participants and setting

The study population remained unchanged, being derived from a suburban General Practice near Glasgow. There were no significant changes within the make up of the practice in the intervening 11 months with the list size remaining constant at 10 000. The inclusion criteria were unchanged, with patients aged 16 or over being eligible to attend the practice chaplain (Stewart-Brown and Janmohamed, 2008).

During the additional 11 months, data was only collected regarding patients attending PCC [including those co-prescribed AD and those not on AD (No-AD)]. Data were not collected for patients on AD that did not attend PCC as this was not required for the objectives of this further study.

Patients were, as before, given verbal and written information at the initial contact with the chaplain regarding the purpose of the evaluation. Written consent to use their data was obtained at this point. A case note review was performed on completion of the data collection, to identify those co-prescribed AD. This allowed the creation of an AD and No-AD group from those attending PCC.

### PCC intervention

Patients presenting to a clinician with symptoms of depression/anxiety, a ‘modern malady’ or psychosocial crisis were offered referral to PCC as one of several treatment options. The decision to refer patients to PCC was based exclusively on the clinicians usual consultation skills with shared management of treatment options being operational (Elwyn *et al*., 2000). Some patients took AD and attended PCC, whilst some attended PCC alone. To the authors knowledge no patient attended additional counselling during the study period.

The practice chaplain typically saw patients within seven working days of referral. Appointment length was up to 1 h with appointment duration and number of follow-up appointments being directed by the patient. The type of intervention offered was based on a fusion of the ‘Human Givens’, ‘Modern Maladies’ and spiritual direction approaches described above.

### Assessment tools

The Warwick Edinburgh mental well-being score (WEMWBS) was used throughout this study to assess pre- and post-PCC well-being. The full rationale for its use is documented previously (Macdonald, 2017a). It is a well-validated 14-item well-being scale marked by the summation of a 1–5 likert scale (Braunholtz *et al*., 2007; Tennant *et al*., 2007; Maheswaran *et al*., 2012; Macdonald, 2017a) Appendix 1. Minimum score is 14 maximum score is 70. An increase of between 3–8 points in WEMWBS is seen as evidence of clinically important and statistically meaningful improvement (Maheswaran *et al*., 2012).

Warwick medical school granted permission for its use in January 2015. The chaplain administered the WEMWBS at the patient’s first appointment and follow-up WEMWBS were sent with stamped addressed envelopes at 6 and 12 weeks.

Appendix 2 shows the integrated demographic and presenting issue(s) questionnaire. This was also administered by the chaplain at the first appointment. The demographic information collected covered the same areas as previous studies in PCC with age, ethnicity and employment status being subdivided into pre-defined categories (with tick box), in the same manner (Mowat *et al*., 2012; Bunniss and Mowat, 2013; Kevern and Hill, 2015). The presenting issue(s) were also derived from the results of previous PCC studies (Mowat *et al*., 2012; Bunniss and Mowat, 2013). This questionnaire was created for the service evaluation; patients could select more than one presenting issue.

Utilisation of GP appointments was assessed before and after attending PCC. This involved screening the patient’s electronic file to determine the number of GP visits in the 12 weeks before and after attending PCC. A judgement was made as to whether these appointments related to mental health issues. This judgement was not based on read codes but rather by scrutinising the consultation entry. The consultation was considered to relate to mental health if symptoms of depression, anxiety or a ‘modern malady’ were identified. Those attending for non-mental health reasons were not counted.

### Data management and analysis

Data were entered into encrypted excel spreadsheets: WEMWBS, demographic details and presenting issue(s). WEMWBS data were handled in line with guidelines (Stewart-Brown and Janmohamed, 2008). Only one missing value was tolerated and given the lowest possible value. If more than one value was missing the data were discarded for that patient. Not all patients completed a 12-week (3^rd^) WEMWBS questionnaire with the consequent creation of the ‘final’ WEMWBS group, where ‘final’=patient’s 3^rd^ WEMWBS and patient’s 2^nd^ WEMWBS in those not completing a 3^rd^ WEMWBS. This was consistent with previous research (Kevern and Hill, 2015; Macdonald, 2017a) and the concept of describing a patient’s 2^nd^ WEMWBS as their ‘final’ WEMWBS seemed to remain reasonable if they did not respond to a 3^rd^ WEMWBS questionnaire. This approach was also helpful (in tightening confidence intervals (CI) and increasing external generalisabilty) given some of the smaller numbers seen in Tables 5 and 7. A third party who was unaware of the study concept checked the data entry. GraphPad Prism version 7 was used to analyse the data from excel.

## Results

**Figure 1:**
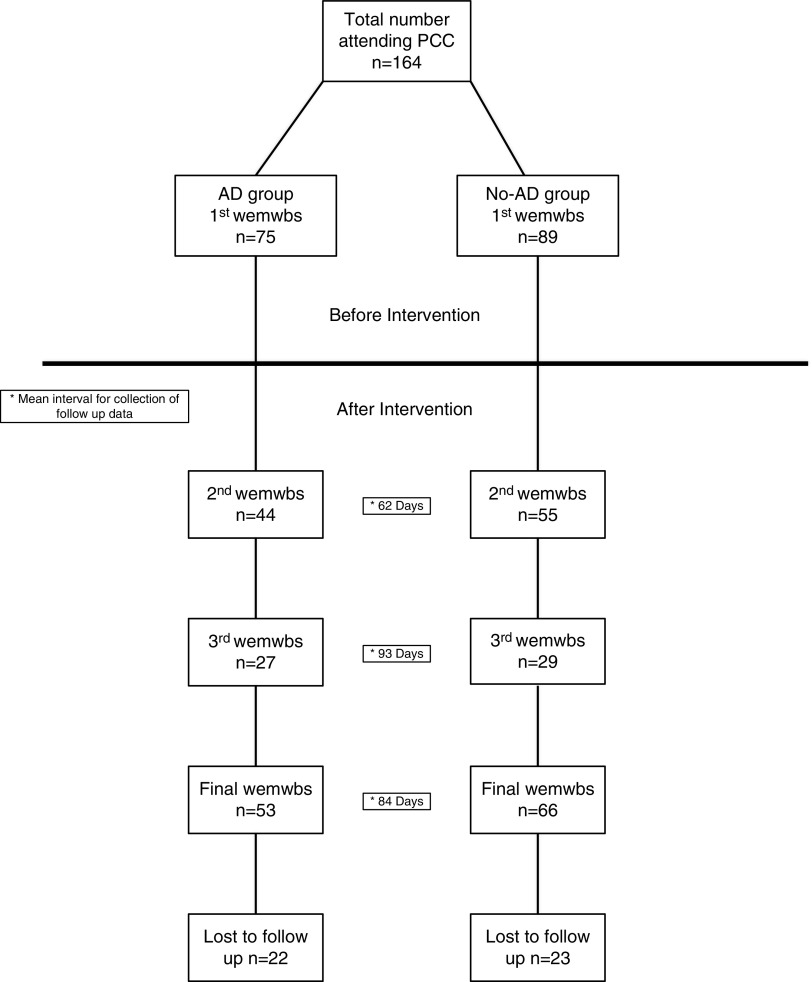
Flowchart of study participants, PCC= primary care chaplaincy; AD= antidepressants; WEMWBS= Warwick–Edinburgh mental well-being score

In total, 164 patients attended PCC during the evaluation; 75 (46%) patients were co-prescribed AD. The mean interval for completion of follow-up WEMWBS data was 2^nd^ (62 days), 3^rd^ (93 days) and Final (84 days). Figure 1 shows a flow chart of study participants.

### Baseline characteristics

**Figure 2:**
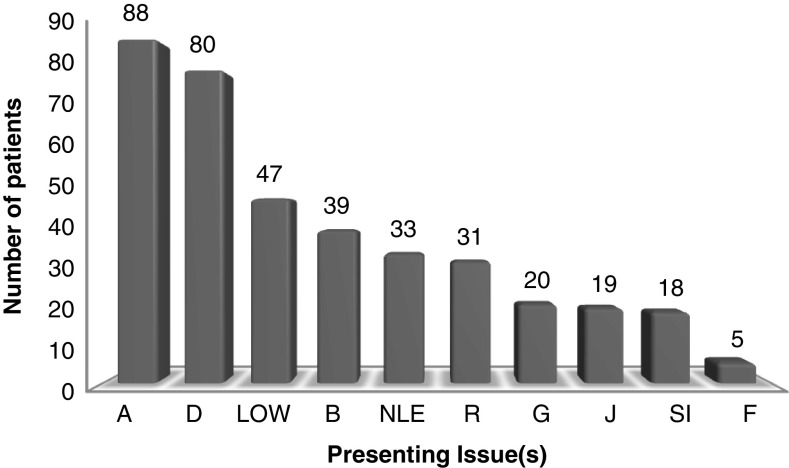
Presenting issue(s) in patients attending primary care chaplaincy. Total numbers shown. D = Depression; A = Anxiety; R = Relationships; J = Job; B Bereavement; SI = Self-image; LOW = Loss of well-being; G = Guilt; NLE = Negative life experience; F = Financial

Socio-demographic data were compared between AD and No-AD groups as shown in Table 1. *χ* ^2^ tests (*χ* ^2^ test for trend in age groups) were used to compare data. There was no statistically significant difference in characteristics between groups:

- Ethnicity: 97.6% of patients were white, with inter-group difference in ethnicity not tested for.
- Gender: there were a higher proportion of females in both groups by a 4:1 ratio.
- Employment status: 50% of participants were in paid employment, with the next most prevalent group being the retired.
- Age: the majority of patients were aged over 40.
- WEMWBS: baseline scores for each demographic are shown in Table 2, with an unpaired *t*-test for sex and one-way analysis of variance (ANOVA) for the remaining categories being used to test for difference.
  The age group with the highest baseline WEMWBS score was the over 65s. This difference was a statistically significant between groups 40–54 and>65s (*P*-value 0.04). Those in paid work or full-time education had higher baseline WEMWBS than those who were out of work or permanently sick or disabled (*P*-value <0.001). Baseline WEMWBS did not differ by sex (*P*-value 0.78).
- Presenting issue(s): Figure 2 shows the prevalence of presenting issue(s) with patients being able to select one or more issue(s). Due to the non-independence of categories statistical testing between AD and No-AD group was not undertaken.
- Baseline WEMWBS by AD use: using an unpaired *t*-test, AD group showed a statistically significantly lower score of 33.6 95% CI 31.6–35.6 than the No-AD group 39.2 95% CI 37.3–41, *P*<0.001.
- Loss to follow-up: *n*(%). AD group 22 (29), No-AD group 23 (26). There was no statistical difference when tested by *χ* ^2^ test (*P*=0.62).

**Table 1:** Socio-demographic status of evaluation participants

	AD group (*n*=75)	no-AD group (*n*=89)	*χ* ^2^ Test of difference
Demographic variableS	*n* (%)	*n* (%)	*P*-value
Sex			
Male	13(17.3)	20(22.5)	*P*=0.41
Female	62(82.7)	69(77.5)	
Age			
16–24	6(8.0)	5(5.6)	*P*=0.81
25–39	18(24.0)	30(33.7)	
40–54	19(25.3)	17(19.1)	
55–64	17(22.7)	18(20.2)	
>65	15(20.0)	19(21.4)	
Employment status			
Full time education	2(2.7)	4(4.5)	*P*=0.78
Not working	4(5.3)	5(5.6)	
Domestic reason out of work	8(10.7)	8(9.0)	
Paid work	37(49.3)	45(50.6)	
Permanently sick or disabled	7(9.3)	5(5.6)	
Retired	17(22.7)	22(24.7)	

%=percentage of each group.

**Table 2:** Baseline Warwick–Edinburgh mental well-being score (WEMWBS) demographic variables

	Average baseline WEMWBS	
Socio-demographic	Mean(SD)	95% CI
Sex		
Male	37(8.5)	33.9–40
Female	36.52(9.3)	34.9–38.1
Age		
16–24	37(7.8)	31.8–42.2
25–39	35.8(7.8)	33.5–38
40–54	34(9.1)	30.9–37.1
55–64	36.9(9.1)	33.8–40.1
>65	40(10.65)	36.3–43.7
Employment status		
Full time education	38(3.2)	34.6–41.4
Not working	35(9.6)	27.6–42.4
Domestic reason out of work	30.8(8.3)	26.4–35.2
Paid work	37.9(7.7)	36.1–39.6
Permanently sick or disabled	26.4(5.3)	23.1–29.7
Retired	39.7(10.6)	36.2–43.1

Mean, standard deviation (SD) 95% confidence interval (CI).

### Summary of baseline characteristics

There was marked similarity between AD and No-AD groups in terms of demographics. The vast majority was white with most patients being female. Half of participants were in paid work. Demographic characteristics affected baseline WEMWBS with age>65 associated with the highest baseline well-being. Being unemployed or too unwell to work had the lowest baseline well-being. The most common presenting issues were depression, anxiety, loss of well-being and bereavement. The AD group had a significantly lower baseline WEMWBS but attrition rates were similar in both groups.

### Impact of presenting issue on baseline and change in WEMWBS

**Figure 3:**
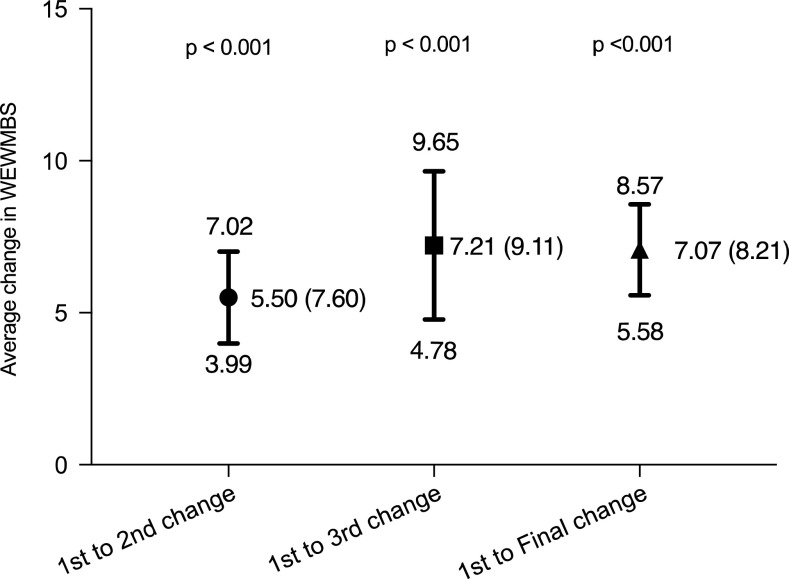
Mean change in Warwick–Edinburgh mental well-being score (WEMWBS) comparing baseline score with 2^nd^, 3^rd^ and final. Mean (SD), 95% confidence interval. 1^st^ to 2^nd^ = change between 1^st^ WEMWBS and 2^nd^ WEMWBS, 1^st^ to 3^rd^ = change between 1^st^ WEMWBS and 3^rd^ WEMWBS and 1^st^ to Final = change between 1^st^ WEMWBS and Final WEWMBS.

- Baseline WEMWBS by presenting issue: the data are presented in Table 3. Those reporting self-image issues or financial concerns had the lowest baseline WEMWBS scores, reflecting poorer well-being scores. Each presenting issue was <40.5 which is the threshold cited as the risk for major depression (Donatella, 2012).
- Change in WEMWBS: 2^nd^, 3^rd^ and final. Follow-up WEMWBS scores were
  divided into three groups: 2^nd^ WEMWBS, 3^rd^ WEMWBS and final WEMWBS as shown in Figure 3. Each of the WEMWBS change scores showed a statistically significant improvement (*P*-value <0.001). Although the final WEMWBS change score was slightly lower than the 3^rd^ WEMWBS change score, this difference was not statistically significant (unpaired *t*-test *P*-value 0.91). It was noticed that the change in final WEMWBS score was likely to be slightly lower than the 3^rd^ WEMWBS category due to the inclusion of some 2^nd^ WEMWBS. There was a mean improvement of 1.71 between the 2^nd^ and 3^rd^ WEMWBS scores; however, when tested by an unpaired *t*-test this difference was not significant (*P*-value 0.21).
- Change in WEMWBS by AD use: each of the groups showed a statistically significant improvement in WEMWBS as seen in Table 4. There was a mean improvement between the 2^nd^ and 3^rd^ WEMWBS in the AD and no-AD groups of 1.5 and 1.88, respectively. Neither was significant with *P*-values of 0.45 and 0.33, respectively. The change in WEMWBS between AD and no-AD groups were compared (by unpaired *t*-test) and no significant differences were observed: 1^st^–2^nd^ *P*=0.79, 1^st^–3^rd^ P=0.99, 1^st^ to final *P*=0.96.
- Change in WEMWBS by presenting issue: the change of WEMWBS score (final) for each presenting issue is presented in Table 5. One-way ANOVA was not possible due to the non-independence of the groups. All presenting issues showed a positive change in WEMWBS score after attending PCC. The improvement in WEMWBS in the majority of presenting issues was similar to the improvement seen in both AD and No-AD groups above. There was a larger improvement noticed in those with loss of well-being and bereavement. It should be noted that Financial concerns and Job, had confidence intervals that crossed zero due to small numbers.

**Table 3:** Baseline Warwick–Edinburgh mental well-being score (WEMWBS) by presenting issue

Presenting issue	Mean (SD)	95% Confidence interval	No. of patients^a^
Relationship	37.71 (7.9)	34.80–40.62	31
Anxiety	36.84 (9.4)	34.86–38.82	88
Negative life event	36.45 (7.7)	33.72–39.19	33
Bereavement	36.13 (8.1)	33.46–38.81	39
Job	35.95 (7.0)	32.56–39.34	19
Loss of well-being	34.53 (9.3)	31.79–37.27	47
Guilt	33.05 (7.9)	29.33–36.77	20
Depression	32.66 (8.8)	30.67–34.65	80
Image	29.44 (7.8)	25.56–33.33	18
Financial concerns	26.4 (4.6)	20.67–32.13	5

a Patients could select more than one presenting issue, thus no test for difference performed.

**Table 4:** Comparison of change in Warwick–Edinburgh mental well-being score (WEMWBS) in antidepressant (AD) group and no-AD group subdivided by timing of WEMWBS score

AD co-prescription	Mean (SD)	95% CI	*P*-value
AD group			
1^st^ to 2^nd^	5.72(6.5)	3.74–7.71	*P*<0.001
1^st^ to 3^rd^	7.22(10.24)	3.17–11.27	*P*<0.001
1^st^ to Final	7.09(8.01)	4.78–9.25	*P*<0.001
No-AD group			
1^st^ to 2^nd^	5.33(8.42)	3.05–7.60	*P*<0.001
1^st^ to 3^rd^	7.21(8.08)	4.13–10.28	*P*<0.001
1^st^ to final	6.94(8.34)	4.89–8.99	*P*<0.001

1^st^ to 2^nd^=change between 1^st^ WEMWBS and 2^nd^ WEMWBS, 1^st^ to 3^rd^=change between 1^st^ WEMWBS and 3^rd^ WEMWBS and 1^st^ to Final=change between 1^st^ WEMWBS and Final WEWMBS. Mean (SD) 95% confidence interval (CI) and *P* value.

**Table 5:** Change in Warwick–Edinburgh mental well-being score (WEMWBS) from baseline to final score by presenting issue

Presenting issue (*n*)	Mean (SD)	95% confidence interval
Depression (56)	6.68 (8.6)	4.37–8.99
Anxiety (59)	7.37 (9.3)	4.95–9.79
Loss of well-being (33)	8.67 (9.3)	5.35–11.98
Bereavement (31)	9 (8.3)	5.96–12.04
Relationship (23)	4.34 (7.9)	0.89–7.7
Job (9)	4.11 (10.9)	−4.33–12.55
Image (11)	7.45 (9.3)	1.15–13.76
Guilt (14)	7.24 (4.6)	4.54–9.88
Negative life event (26)	6.38 (5.0)	4.35–8.41
Financial concerns (2)	5.00 (7.0)	−58.53–68.53

## Summary

PCC was associated with an improvement in both 2^nd^ and 3^rd^ WEMWBS but this was most marked at the 2^nd^ score with a slight plateauing at the 3^rd^ score. Those with poor self-image or financial concerns had the lowest baseline WEMWBS. PCC was associated with a similar improvement in WEMWBS of approximately 7 in both AD and No-AD groups. Eight of the ten presenting issues showed an improvement in well-being. Those with issues of bereavement or loss of well-being showed the greatest improvement in WEMWBS.

### Impact of having single or multiple presenting issues on baseline and change in well-being

Most patients had more than one presenting issue *n*= (105). No patients had more than five presenting issues. The number of patients with follow-up data by number of presenting issues (PI) was: 1 PI *n*= 42, 2 PI *n*= 34, 3 PI *n*= 26, 4 PI *n*= 9, 5 PI *n*=8 and a ‘multiple’ group of>1 PI *n*=77.

- Baseline WEMWBS: unpaired *t*-tests were used to compare single and multiple issue presentations, as shown in Table 6. Those with multiple issues in the AD group showed a statistically significantly lower baseline WEMWBS than those with a single-issue presentation (*P*=0.02). There was no difference between single and multiple issue presentations in the No-AD group (*P*=0.19).
- Change in WEMWBS: unpaired *t*-tests were used to compare single and multiple issue presentations, as shown in Table 6. Whilst single issue presentations had slightly higher change scores in both groups this was not significant in either AD (*P*=0.49) or No-AD (*P*=0.57) groups.

**Table 6:** Baseline and change in Warwick–Edinburgh mental well-being score (WEMWBS) in single issue and complex presentations: antidepressant (AD) group and no-AD group

AD co-prescription	Mean (SD)	95% CI	*P*-value
AD group			
Single issue (baseline)	38.4(9)	33.8–43.1	
Single issue (change)	8.35(6.5)	5.0–10.69	*P*<0.001
Multiple issues (baseline)	32.5(8.3)	29.7–35.3	
Multiple issues (change)	6.72(8.8)	3.7–9.7	*P*<0.001
No-AD group			
Single issue (baseline)	41.8(8.7)	38.2–45.4	
Single issue (change)	7.68(8.8)	4.05–11.30	*P*<0.001
Multiple issues (baseline)	39(8.3)	36.4–41.6	
Multiple issues (change)	6.48(8.3)	3.9–9.1	*P*<0.001

CI=confidence interval. *P* values calculated by paired *t*–test.

This finding was further tested as follows in Table 7. One-way ANOVA tests were run for both AD and No-AD groups. No significant difference was found within each group’s baseline WEMWBS score and no linear trend was found in either group. However, there was a significant difference (unpaired *t*-test, *P*=0.03) comparing those with two presenting issues between the AD and No-AD group. One-way ANOVA was repeated for change in WEMWBS scores and no significant difference was found within each group or between groups.

**Table 7:** Baseline and change in Warwick–Edinburgh mental well-being score (WEMWBS) by number of presentations: antidepressant (AD) group and no-AD group

Number of issues	AD group			No-AD group		
Baseline WEMWBS						
One issue	38.4(9)	33.8–43.1		41.8(8.7)	38.2–45.4	
Two issues	31.8(8.9)	26.6–36.9		41.8(7.4)	38.3–45.2	
Three issues	35.8(6.2)	31.8–39.7		36.3(9.5)	30.8–41.8	
Four issues	27.3(9.5)	17.3–37.3		36.3(5.6)	22.2–50.4	
Five issues	34.4(7.8)	24.7–44.1		33(3)	25.5–40.5	
Change WEMWBS						
One issue	8.35(6.5)	5.0–10.69	*P*<0.001	7.68(8.8)	4.05–11.30	*P*<0.001
Two issues	5.3(4.7)	2.7–7.9	*P*=0.001	5.2(7.9)	1.5–8.9	*P*=0.008
Three issues	7.5(11.7)	0.07–14.9	*P*=0.04	8.7(9.1)	3.5–13.9	*P*=0.003
Four issues	12(11.3)	0.15–23.9	*P*=0.04	7(8)	−12.8–26.8	*P*=0.27
Five issues	5(7.4)	−4.1–14.1	*P*=0.20	7.7(4.7)	−4.1–19.4	*P*=0.11

Mean(SD) 95% confidence interval. *P* values calculated by paired *t*-test.

## Summary

Baseline WEMWBS was unaffected by the number of presenting issues in those not on AD. There was a significantly lower baseline WEMWBS in those with multiple issues on AD. However, this difference was not sustained when those with multiple presenting issues were further stratified as in Table 7. Change in WEMWBS ranged from 6.48 to 8.35 being clinically significant in those with single and multiple issues. There was no significant difference in improvement in those with single issues relative to those with multiple issues. Change in WEMWBS score was not affected by the number of presenting issues in either AD or No-AD group. The level of improvement remained significant until three issues were reached in the No-AD group and four issues in the AD group.

### Impact of PCC on appointments

Wilcoxon Signed Rank tests were used to test for difference in the number of pre- and post-PCC GP appointments. The data are presented in Table 8. There was a significant reduction in GP appointment utilisation in the three months after attending PCC. A statistically significant difference was observed in the No-AD group but not those in the AD group.

**Table 8:** Mean number of GP appointments before and after attending primary care chaplaincy (PCC)

	Number of appointments before PCC		Number of appointments after PCC		
Patients status by use of antidepressants	Mean (SD)	95% CI	Mean (SD)	95% CI	*P* value
All *n*=164	1.44 (1)	1.26–1.62	0.92 (1.17)	0.71–1.14	<0.001
AD status					
AD group *n*=75	1.75 (1.11)	1.45–2.06	1.43 (1.43)	1.04–1.83	0.06
No-AD group *n*=89	1.18 (0.82)	0.97–1.39	0.50 (0.69)	0.34–0.68	<0.001

CI=confidence interval. Difference tested for by Wilcoxon Signed Rank Test.

## Discussion

### Comparison with other studies

This study shows marked ethnic homogeneity which contrasts with some PCC studies (Kevern and Hill, 2015) but builds on others (Macdonald, 2017a). There is a marked preponderance of females in keeping with other mental health population studies (Van Der Heyden *et al*., 2009). Well-being is seen to improve to a similar level as other studies in those attending PCC (Kevern and Hill, 2015). GP workload was seen to reduce, again reflecting recent research (Macdonald, 2017b).

### Principal findings

It is noteworthy that PCC was associated with a clinically important (Maheswaran *et al*., 2012) improvement in nearly all presenting issues and that each of these presenting issues had a baseline well-being placing participants at risk of major depression. This suggests that PCC is relevant to the many and varied presentations seen in primary care. The greatest improvements were seen in the loss of well-being and bereavement categories. Loss of well-being reflects the typical undifferentiated presentations seen in primary care with patients often finding it difficult to express their specific symptoms. It is helpful to see that PCC is associated with such positive improvements in well-being in these ill-defined presentations so prevalent in current primary care. It seems clear that those with undifferentiated illness and a diversity of presentations are accessing PCC. Furthermore, PCC seems to be associated with improved well-being in both such groups.

Most patients in this study (64%) had more than one presenting issue, which increases its generalisability given the prevalence of multimorbidity in primary care (Barnett *et al*., 2012). The baseline well-being of patients in both AD and No-AD groups was not significantly affected by the number of presenting issues. This may seem at variance with the expectation that well-being would decline as the number of presenting issues accumulate. However, it may simply reinforce the undifferentiated way in which attendees express their issues, some citing multiple issues with less discrimination than those citing single issues. PCC has also been shown to effective irrespective of the number of presenting issues. It is associated with a clinically significant improvement in well-being when PCC is used is the sole intervention. This was seen in both single and multiple issue presentations. This appears to validate the place of PCC, as an independent therapy, in treating complex illness as these multimorbid patients require their person-centered needs, including those of a spiritual or existential nature, to be addressed (Mercer *et al*., 2009). This emerging evidence base further justifies the inclusion of PCC in the extended primary care team as PCC appears to engage with complexity, undifferentiated illness and whole-person care, each of which are foundational to the medical generalism we seek in current primary healthcare (Howe, 2012).

This study adds novel data regarding participants’ well-being in the context of their demographic status. Approximately 25% of attendees were retired. It is also noted that those over the age of 65 had highest baseline well-being scores. Being elderly is a known risk factor for social isolation (Dury, 2014) affecting up to 30% of the elderly (Landeiro *et al*., 2017). It may be reasonable to postulate that this is why despite having the highest baseline well-being the elderly make such significant use of PCC (particularly if not on AD). It is perhaps unsurprising that those who were out of work or permanently sick/disabled had the lowest well-being scores in the employment category. Combining these groups accounts for nearly 20% of attendees. It is known that unemployment (Brand, 2015) and disability (Tough *et al*., 2017) can both be associated with social isolation . It therefore seems possible that these patient’s low well-being may reflect their isolation and contribute to their presentation to PCC. The four most prevalent presenting issues to PCC were: depression, anxiety, loss of well-being and bereavement. Each of these is known to be associated with social isolation and loneliness (Cornwell and Waite, 2009; Weiss *et al*., 2013; Matthews *et al*., 2016). ANOVA was not carried out for presenting issues due to non-independence of categories. However, it remains evident that categories of finance and self-image showed the lowest baseline WEMWBS scores. This would fit with evidence highlighting the effect of social isolation on self-image/esteem (Hall-Lande *et al*., 2007) and may explain the reduced available social network resulting in use of PCC. Bereavement, itself a recognised risk factor for social isolation, was associated with the largest improvement in well-being. This is of particular note given it was a presenting factor in at least 25% of attendees in this study and >30% in other studies of PCC (Mowat *et al*., 2012; Mcsherry *et al*., 2016). It seems that many users of PCC in this study displayed risk factors for loneliness and social isolation. Social isolation can function as a modern malady, affecting spiritual well-being (Miller, 1985) and increasing consulting rates. It will be important for both referring clinicians and chaplains alike to be mindful of the demographics and presentations that may be indicative of isolation impacting on spiritual well-being. This may impact on clinician’s referral patterns. PCC offers specific features such as longer appointment length and personal continuity that may foster belonging, reduce loneliness and enhance spiritual well-being.

PCC was associated with a reduction in follow up GP appointments in those not on AD. This has not been shown before. One previous study showed no change in GP appointment utilisation but it’s the authors did not specify if non-mental health consultations were excluded (Kevern & Ladbury, 2015). The other previous study did show a reduction in GP appointment utilisation (Macdonald, 2017a). However, this study compared prospective GP appointment utlisation in those attending PCC (not on AD) with those on AD not attending PCC. Clearly those on AD will require GP supervision, which partly explains the lower number of GP appointments in those attending PCC not on AD. However, the current study shows those attending PCC (not on AD) make less use of GP appointments after attending PCC relative to their use prior to attending PCC. This highlights the value of PCC as a sole therapy: it is not only associated with an improved well-being, but it also associated with a reduction in workload. This would seem to be advantageous in the current climate of primary care in the United Kingdom.

### Strengths of this study

The impact of PCC on well-being seems in line with recent research. The objectives of the study have been met with PCC being shown to be utilised by those with undifferentiated illness and risk factors for social isolation. This is thought to be the first PCC study assessing the impact of the type and number of presenting issues on outcome. The effect on GP workload has been further clarified by showing a reduction in GP appointments in those attending PCC.

### Limitations of the study

As with any observational study there is a risk of both bias and confounding. Attempts were made to reduce these by comparing demographics between the AD and No-AD group with no difference found. Due to the non-independence of the presenting issues categories differences between the AD and No-AD group were not calculated. As noted in the initial study (Macdonald, 2017a) selection bias may be introduced in that patients opting for PCC may be more likely to respond to this than those taking AD. Confounding may occur due to an unequal distribution of negative life events between AD and No-AD groups and indeed the differing types and numbers of presenting issues. Statistical difference was unable to be tested for in both baseline and change well-being scores for presenting issues, again due to non-independence of categories. Whilst this would have been preferable it was still felt that there were useful observations from the data due to reasonable participant numbers. There were some data with confidence intervals crossing zero, however these were not utilised in the analysis. Finally, the follow-up time period for assessing workload was relatively short at three months.

## Conclusion

This study further evidences the association of PCC (as the sole intervention) with improved well-being. Patients presenting with undifferentiated and complex illness are also utilising PCC. PCC seems to be associated with an improved well-being irrespective of type or number of presenting issue(s), being responsive to those with both undifferentiated and complex illness. Those with risk factors for social isolation and loneliness are accessing PCC. GP consultation rates appear to reduce when PCC is used. It does appear that PCC is indeed able to respond to the needs of our increasingly isolated populations who present with non-specific and varied problems. There remain opportunities for further research, which should include weighting of presenting issues to clarify which are most responsive to PCC. A prospective randomised controlled trial will ultimately be the ideal methodology. That said this study adds to the growing evidence base for the place of spiritual care in our current societal context. PCC is functioning as a point of refuge for those with modern maladies arising from isolation in addition to those with complex undifferentiated well-being issues. PCC therefore seems to sit well in the current political and pragmatic landscape of primary care.
